# Structural and Drug Targeting Insights on Mutant p53

**DOI:** 10.3390/cancers13133344

**Published:** 2021-07-03

**Authors:** Ana Sara Gomes, Helena Ramos, Alberto Inga, Emília Sousa, Lucília Saraiva

**Affiliations:** 1 LAQV/REQUIMTE, Laboratório de Microbiologia, Departamento de Ciências Biológicas, Faculdade de Farmácia, Universidade do Porto, 4050-313 Porto, Portugal; anasarag4@gmail.com (A.S.G.); helenainrr@gmail.com (H.R.); 2 Laboratory of Transcriptional Networks, Department CIBIO, University of Trento, Via Sommarive 9, 38123 Trento, Italy; alberto.inga@unitn.it; 3 Laboratory of Organic and Pharmaceutical Chemistry, Department of Chemical Sciences, Faculty of Pharmacy, University of Porto, Rua de Jorge Viterbo Ferreira, 228, 4050-313 Porto, Portugal; esousa@ff.up.pt; 4 CIIMAR-Interdisciplinary Centre of Marine and Environmental Research, University of Porto, Novo Edifício do Terminal de Cruzeiros do Porto de Leixões, Avenida General Norton de Matos, S/N, 4450-208 Matosinhos, Portugal

**Keywords:** mutant p53, reactivators, targeted anticancer drugs, transcription factor

## Abstract

**Simple Summary:**

The tumor suppressor protein p53 is frequently mutated in human cancers, and its reactivation represents an encouraging hope for precision anticancer therapy. Despite this, the clinical use of compounds capable of restoring the wild-type-like function to mutant p53 (mutp53) is not yet a reality. A more detailed understanding of the structural features of p53 mutations and the molecular mechanisms of mutp53 reactivators is critical to drive an efficient translation of these compounds into the clinic. In addition, this knowledge will provide insights into wild-type and mutp53 pharmacology at molecular levels, fostering the design and development of improved mutp53-targeted therapies to halt cancer.

**Abstract:**

p53 is a transcription factor with a pivotal role in cell homeostasis and fate. Its impairment is a major event in tumor onset and development. In fact, about half of human cancers bear *TP53* mutations that not only halt the normal function of p53, but also may acquire oncogenic gain of functions that favor tumorigenesis. Although considered undruggable for a long time, evidence has proven the capability of many compounds to restore a wild-type (wt)-like function to mutant p53 (mutp53). However, they have not reached the clinic to date. Structural studies have strongly contributed to the knowledge about p53 structure, stability, dynamics, function, and regulation. Importantly, they have afforded relevant insights into wt and mutp53 pharmacology at molecular levels, fostering the design and development of p53-targeted anticancer therapies. Herein, we provide an integrated view of mutp53 regulation, particularly focusing on mutp53 structural traits and on targeting agents capable of its reactivation, including their biological, biochemical and biophysical features. With this, we expect to pave the way for the development of improved small molecules that may advance precision cancer therapy by targeting p53.

## 1. Introduction

**Figure 1:**
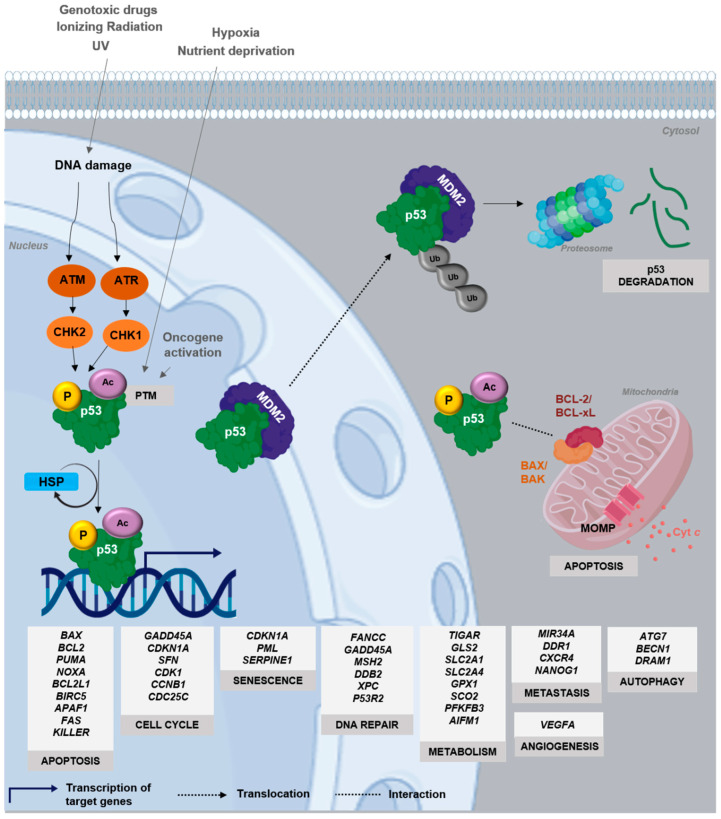
p53 signaling pathway. In unstressed conditions, p53 has a short half-life, being normally kept at low levels by MDM2, an E3 ubiquitin ligase that is itself a transcriptional target of p53, inhibiting p53 transcriptional activity and targeting it for proteasomal degradation via polyubiquitination (Ub). p53 is activated by stress stimuli, such as DNA damage, which activate the upstream kinases ataxia telangiectasia mutated (ATM) and ataxia telangiectasia and Rad3-related protein (ATR), leading to p53 phosphorylation by checkpoint kinase (CHK)1 and CHK2. Other cellular stresses include hypoxia, nutrient deprivation, or oncogene activation, which dislocate p53 from the regulation of inhibitors (e.g., MDM2) through post-translational modifications (PTM), like phosphorylation (P) and acetylation (Ac), inducing stabilization, rapid accumulation, and activation of p53. Molecular chaperones, including Heat Shock Proteins (HSPs), through transient cyclic bind-unwind-release reactions, assist p53 to adopt its proper tertiary and quaternary structure and carry out its tumor suppressor activities. Depending on the stimulus, cell context, or PTM pattern, active p53 transcriptionally regulates the expression of several target genes involved in the control of major cellular processes, including cell cycle, apoptosis, DNA repair, autophagy, senescence, metabolism, metastasis, and angiogenesis. p53 also plays transcription-independent mitochondrial roles, as it can directly bind to BAX/BAK, resulting in their activation and initiation of apoptosis; p53 can also bind to BCL-2/BCL-xL, inhibiting their anti-apoptotic functions, through the disruption of inhibitory complexes between BCL-xL/BCL-2 with BAK/BAX, which results in mitochondrial outer membrane permeabilization (MOMP), cytochrome *c* (cyt *c*) release, and subsequent apoptosis.

The p53 protein belongs to a family of transcription factors, alongside p63 and p73, whose full activity is essential for the prevention of tumor onset and development. p53 is the central hub of a complex and intricate network, regulating major signaling and cell fate decision pathways. It senses diverse physical or chemical distress cellular signals, such as DNA damage, that eventually lead to its activation, stabilization, and accumulation in the cell [1] (Figure 1). Once active, p53 transcriptionally regulates many genes involved in major cellular processes, including cell cycle arrest, senescence, DNA damage repair, metabolic adaptation, and apoptosis [2] (Figure 1). Importantly, p53 activity is tightly controlled by a complex feedback-regulated network involving endogenous negative regulators (e.g., Murine Double Minute 2, MDM2) as well as by post-translational modifications (PTMs) and by interaction with distinct signaling proteins [3,4,5] (Figure 1).

The cell fate specified by p53 activation is context- and tissue-dependent, being mainly related to the nature of cellular stress [1]. In case of mild DNA damage, p53 drives cell cycle arrest for DNA repair, while under severe and irreparable DNA damage, p53 stimulates apoptotic or senescence programs. Other crucial factors, including the expression levels and PTM status of p53, cellular localization, co-factor enrollment, and the architecture of the promoters of target genes, also dictate the type of p53-mediated cellular responses based on the transcription of different subsets of p53 effectors [6,7].

The impairment of p53 tumor suppressor functions, either by mutations in the *TP53* gene (which encodes the p53 protein) or by other abnormalities in the p53 pathway, is a common event among human cancers. Therefore, targeting p53 constitutes an appealing anticancer therapeutic strategy. However, since its discovery, p53 has represented a challenging target for drug discovery, being frequently considered as “undruggable”. In fact, wild-type (wt) p53 and most p53 mutant (mutp53) forms lack binding pockets or allosteric sites, other than the DNA-binding groove itself, hindering rational drug design [8]. Importantly, over 2000 mutp53 forms can be found in human tumors, with differences in their structure, stability, and biological functions, making it virtually impossible to target all of them with a single drug [9,10]. Nonetheless, in vitro and in vivo evidence has proven that restoration of mutp53, with re-establishment of wt-like activity, elicits impressive tumor regression and cell death [11,12,13,14].

This review addresses the most relevant achievements in the functional and structural elucidation of mutp53 as well as of the mutp53-targeting agents reported to date. With this systematization, we expect to bring new insights into mutp53 pharmacology, aiming at the development of improved molecules capable of restoring wt-like properties to mutp53.

## 2. p53 Structure and DNA Recognition

Structural studies with full-length p53 are challenging. Indeed, its flexibility, labile stability, and proneness to aggregation have hampered crystallographic studies. Its molecular weight represents a limitation for studies using conventional nuclear magnetic resonance (NMR) spectroscopy. On the other hand, p53 size is at the limits for cryo-electron microscopy (EM) methodology [15]. To overcome these limitations, a combined multi-technique approach was followed to construct a jigsaw from individual domain data. For this, specific recombinant p53 domains were produced by bioengineering techniques and analyzed by X-ray crystallography and NMR spectroscopy. In addition, some studies to check the spatial 3D arrangement of folded domains were pursued with the full-length protein, using suitable methodologies such as small angle X-ray scattering (SAXS), cryo-EM, and fluorescence resonance energy transfer (FRET) [15,16]. NMR spectroscopy proved to be important for understanding protein-protein and protein-DNA interactions in solution [17]. To complement these studies, electrophoretic mobility shift assay [18], protein microarrays, transactivation assays, fluorescence anisotropy, and isothermal calorimetric [18] titrations, among others, have also been used to unveil binding features and to characterize affinities for DNA and other proteins, peptides, or small molecules [17,19,20,21,22]. Recombinant p53 proteins with specific mutations (naturally occurring alone or combined with second-site mutations that can rescue DNA binding ability) have also contributed to the study of the relevance of specific amino acid residues in p53 stability and activity [23,24,25,26]. Furthermore, computational studies, such as molecular superposition and dynamics, have been used for predictive and comparison purposes in the structure-function relationship [15].

**Figure 2:**
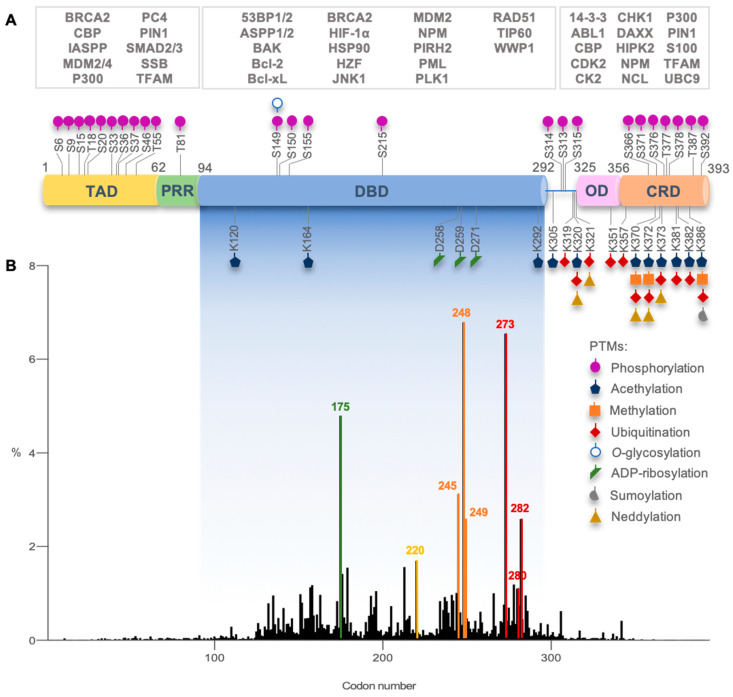
p53 structural domains with overview of post-translational modifications, interacting proteins, and most frequent *TP53* missense mutations in human cancers. (**A**) Each p53 domain (transactivation domain, TAD; proline-rich region, PRR; DNA-binding domain, DBD; oligomerization domain, OD; *C*-terminal regulatory domain, CRD) is target of different post-translational modifications (PTMs; alone or concomitant) that regulate p53 function. p53 interacts directly with different PTM enzymes, transcription co-factors, and other nuclear or cytoplasmic proteins (some examples are depicted in the white boxes, being associated with a p53 interacting domain) that modulate p53 function. Image based on [27,28,29,30]. (**B**) Graphic depicting *TP53* missense mutations relative frequency per codon (DBD encoding codons are highlighted in fading blue box, residues 94–292). Highlighted in green, yellow, orange, red, and dark-red are codons 175, 245, 248, 249, 273, and 282, hotspots for the highest incidence, and codons 280 and 220. These codons code for critical amino acid residues for p53 structure and function. (Graph constructed with GraphPad 7 and data from IARC *TP53* Database, R20, July 2019 [31]).

Active p53 is a homotetramer of four identical chains of 393 amino acids. Each monomer presents a modular structure divided into different domains: an acidic disordered *N*-terminal region, comprising the transactivation domain (TAD) and the proline-rich region (PRR); a central core sequence-specific DNA-binding domain (DBD); a *C*-terminal region, encompassing an oligomerization domain (OD) and a disordered regulatory domain (CRD) [15] (Figure 2).

**Figure 3:**
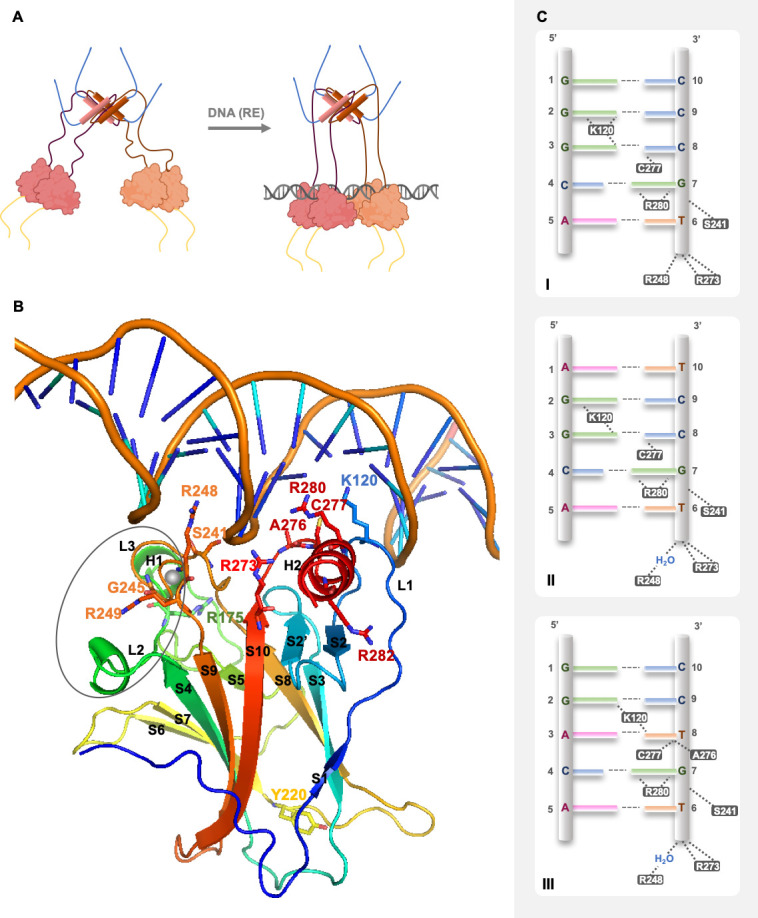
Representation of p53 quaternary structure in solution, structural insights on p53 DBD binding to DNA, and p53 specific DNA-binding modes. (**A**) Spatial conformations of open unbound and closed/compacted bound p53 tetramer to DNA response element (RE) sequence. Pink and orange protein-like cartoons represent the DBDs (each color for one dimer), and sticks represent the respective ODs. The disordered *N*-terminal and *C*-terminal are depicted in yellow and blue, respectively. Representation is based on NMR, SAXS, and EM experimental studies [17,32,33]. (**B**) The 3D model of the DBD binding to cognate DNA sequence (PDB ID code: 1TUP, [42]) highlights the structural organization in helixes, loops, and strands in the *β*-sandwich. Furthermore, it depicts the amino acid residues involved in DNA recognition, establishing interactions with DNA, specifically K120 (L1), S241 and R248 (L3), R273 (S10), A276, C277, and R280 (H2). Other residues are also key partners for the correct binding positioning or structural stability, such as R175 (L2), Y220 (S7–S8 turn), G245 (L3), R249 (L3), and R282 (H2). Other regions of the DBD that are important for the assembly in dimers, namely H1, L2, and L3, are highlighted by a gray ellipse. Image created using PyMOL software [44]. H—helix; L—loop; S—strand. Image based on [15]. (**C**) p53 binding to three pentameric DNA sequences belonging to decameric half-sites of the consensus region of p53 target genes. The side chain of residue R280 is a steady point of contact between p53 and DNA, making two invariant hydrogen bonds to the conserved guanine base (G7) in the DNA major groove. The residue R273 anchors p53 to DNA backbone phosphates (grey pipe) in the central region of each half-site. The side chain of residue S241 also makes invariant contacts to DNA backbone phosphates. The specific interactions established by residues K120, A276, and C277 dramatically change when the base pair, at positions 3 and 8 of the DNA half-site, is changed from G/C to A/T. When in presence of the G/C base pair, the residue K120 makes three (**I**) or two (**II**) hydrogen-bonds with successive guanine bases (G2 and G3); when in presence of A/T base pair (**III**), it establishes interactions with G2 and T8. Regarding positions 3 and 8, the residue C277 interacts with C8 in the case of the G/C base pair (**I**,**II**); in the case of the A/T base pair (**III**), it establishes van der Waals interactions with T8. The most marked binding difference is observed with the residue A276: the interaction between its side chain and the DNA base only occurs with the A/T base pair (**III**), with a hydrophobic interaction occurring among the methyl groups of A276 and T8. The doublet central DNA half-site A/T base pairs (positions 5 and 6) are crucial in water-mediated interactions, playing an essential role in the cooperative binding of the core dimer to its DNA half-site through minor groove hydration. This interaction is directly mediated by the side chain of residue R248 (**I**) or indirectly via water molecules (**II**,**III**). The base pairs at positions 1 and 10 do not interfere with protein-DNA complex stability. Image based on [36,45].

In solution, using SAXS, NMR, and EM techniques, it was possible to understand that full-length p53 forms a tetramer with an opened, cross-shaped structure, having the ODs at its center and a pair of loosely coupled DBD dimers at the ends, which are accessible to bind to cognate DNA and partner proteins. Upon DNA binding, the structure closes around DNA and becomes more compact [17,32,33] (Figure 3A). p53 is a biologically active transcription factor as a homotetramer (assembled as a dimer of dimers; p53 dimers are formed co-translationally [34]), and DNA recognition occurs upon a cooperative binding process [35]. This means that the formation of DBD-DBD dimers interaction followed by the tetramerization through the ODs is favored in the presence of DNA, and both protein-protein and protein-DNA interactions contribute to the overall complex stability [36].

p53 exerts its transcriptional activity through recognition of a short specific DNA sequence in the target genes’ promoters, called the response element (RE) [36,37]. The REs enable a coordinated and flexible response, given the existence of many variations of the consensus sequence. Thus, upon stress stimuli, p53 can bind to multiple scattered REs in the genome, eliciting the transcription of sets of genes and related cellular responses [38]. The p53 target RE binding site consists of two decameric motifs (half-sites) with the consensus sequence RRRCWWGYYY (R = A, G; W = A, T; Y = C, T); C4 and G7 positions should be conserved among the variant sequences [39,40]. The degenerate nature of the consensus already exemplifies why there are many variant sequences of REs among the different p53 target genes. In addition, mismatches from the consensus are frequent among established p53 REs at target gene promoters. Thus, there is a wide range in sequence-specific binding affinities, and this is correlated with variations in the protein-DNA contact geometry, providing insights into the mechanism of p53 function and regulation and the potential to elicit gene expression changes that are tailored to specific stress conditions through modulation of nuclear p53 protein levels or by specific PTMs or protein cofactors [36,41].

In 1994, Cho et al. reported for the first time the crystallographic model of p53 DBD with DNA at 2.2 Å [42]. The p53 DBD is an immunoglobulin-like central *β*-sandwich of two antiparallel *β*-sheets, providing the basic scaffold for the DNA-binding surface (Figure 3B). This surface is constituted by two large loops, L2 and L3, the latter intercalated by a short helix, H1, stabilized by a tetrahedral coordination of two residues of each loop to a zinc atom (L2–C176, H179; L3–C238, C242) and by a loop-sheet-helix (LSH) motif constituted by L1, *β*-strands S2 and S2′, the end of *β*-strand S10 and *C*-terminal of H2 (Figure 3B).

The LSH motif makes specific contacts with the major groove of the target DNA, through hydrogen bonds, salt-bridges, and structural water molecules in a tight network of hydrogen bonds that ensures the correct orientation of each amino acid residue. In the DNA major groove, the side chains of residues K120 (L1), A276, C277, and R280 (H2) interact with DNA bases via hydrogen bonds, and the side chain of residue R273 (S10) and the backbone amide of residues K120 and A276 establish interactions with the DNA backbone phosphates via salt bridge interactions (Figure 3B,C). The L3 contains the residues S241 and R248, which have contact with DNA backbone phosphates in the DNA minor groove; R248 is essential for DNA recognition (Figure 3B,C) [42,43]. Other residues also play an important role in stabilizing the p53 DNA-binding surface, such as R175 (L2), G245 (L3), R249 (L3), and R282 (H2) [42,43] (Figure 3B,C). The finer details of DNA-contact geometry show an interesting variation that depends on the DNA sequence [36]. A description of the known modes of binding of each p53 residue to the DNA consensus region was provided by Kytainer et al. [36] (Figure 3C).

Each half-site is recognized by a symmetrical dimer of two p53 DBDs with a relatively small, self-complementary protein-protein interface with around 600 Å^2^ of buried surface area [26]. The interface is stabilized by hydrophobic, water-mediated polar interactions and hydrogen bonds between the zinc regions, involving H1, L2, and L3, of the two p53 DBDs (Figure 3B). There are two buried water molecules that provide an internal hydrogen-bonding network as a central anchor, linking the two zinc regions, which support H1 and L3 configuration. A nonpolar interaction shell is formed by surface residues from H1 and L3 hairpin via residues P177, H178, M243, and G244. Next to this hydrophobic area, there are two hydrophilic networks that contribute to its stability, one far from DNA, involving charged residues from the two monomers (specifically R174, E180, R181, and several water molecules), and the second one near to DNA, with a first-shell hydration of the protein surface facing DNA, via N239, S241, and R248 side chains [42].

The interaction of protein-DNA is the ultimate contribution to dimer stabilization. Dimer protein-protein interactions may also contribute to DBD integrity, considering the bidentated salt bridges between the polar and charged surface residues D184 with R175, and R249 with E171 on opposite sides of each monomer [36,46]. Kantarci et al., in addition to acknowledging that both p53 DBD monomers in the dimer establish the same interactions with the DNA RE, advanced the hypothesis that, in a dimer, the p53 DBD interactions with DNA were stronger for one monomer than for the other [47]. In fact, this has also been supported by the spatial rearrangement of the tetramer complexed with DNA verified in cryo-EM works [17,32,33].

## 3. Dynamics and Regulation of p53

Although intrinsic domain disorder hampers p53 studies, it allows a structural plasticity, reflected in binding promiscuity observed by p53 interaction with a wide range of target DNA sequences and interacting proteins [45] (Figure 2A and Figure 3C). Additionally, intrinsic disorder is directly correlated with low thermodynamic and kinetic stability: in fact, p53 half-life time at body temperature is about 9 min, with rapid cycles between folded and unfolded states [19,48,49]. These features have been postulated as an evolutionary advantage, providing a tight regulation of functionally active p53 cellular levels [45]. Additionally, in normal cells, under no stress conditions, p53 levels are low, as it is negatively regulated by different proteins, including its major endogenous inhibitor MDM2 (Figure 1). This protein not only prevents DNA recognition by directly binding to p53, but also targets p53 for proteasomal degradation via polyubiquitination or nuclear export via monoubiquitination by its E3 ligase activity. Under stress stimuli, p53 is displaced from MDM2 negative regulation, with subsequent stabilization and activation of its transcriptional functions [50] (Figure 1). Furthermore, heat shock proteins (HSPs), such as HSP40, HSP70, and HSP90, act like chaperones, transiently binding to free p53 and promoting its correct folding upon their dissociation for DNA RE binding [28,51,52,53] (Figure 1).

Besides p53 protein stabilization, several other factors seem to reinforce the fine tuning of stimuli-dependent p53 regulation and cell fate decision, including the protein level dynamics, cellular localization, and PTMs [54] (Figure 1 and Figure 2A). Methylated or ubiquitinated p53 correlates with unstressed cells. In contrast, phosphorylation, acetylation, neddylation, or sumoylation are more frequent in cells upon DNA damage, increasing p53 DNA-binding affinity to target genes [29,55,56]. One PTM may influence the occurrence of another, unlocking additional layers of regulation, affecting protein stability, function, protein-protein interaction, and the recruitment of transcriptional co-factors and machinery, favoring DNA-binding toward specific target genes [57,58].

## 4. Mutant p53 Functions in Tumorigenesis

*TP53* mutations occur in more than half of human cancers, with colorectal, head and neck, esophagus, female genital organs, and lung cancers exhibiting the highest prevalence (37–43%; International Agency for Research on Cancer (IARC) TP53 Database, R20, July 2019) [31]. *TP53* mutations occur in both germline (associated with Li-Fraumeni syndrome) and sporadic contexts and can be found throughout the whole gene [31] (Figure 2B). Missense mutations are the most frequent alterations in *TP53* (over 75%) and mainly occur in the DBD region (over 80%) [59]. In fact, in the p53 DBD, there are six hotspot missense mutations in codons 175, 245, 248, 249, 273, and 282, with high clinical significance [31] (Figure 2B). As previously mentioned, these codons code for residues with important roles in p53 structure and function.

**Figure 4:**
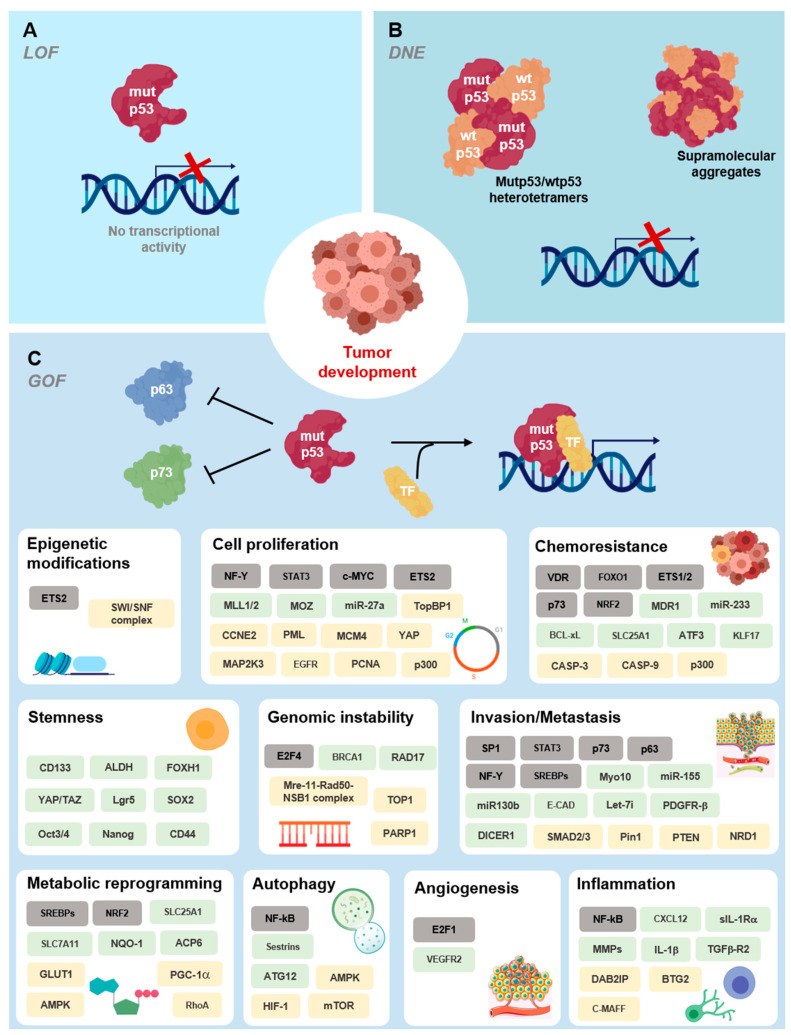
Functional activities of mutp53 in cancer. (**A**) In general, p53 mutations lead to loss of DNA-binding ability and impairment of the p53 response (loss of function, LOF). (**B**) A dominant negative effect [18] of mutp53 over wtp53 occurs through the formation of heterotetramers and supramolecular aggregates with wtp53. (**C**) Mutp53 gain of function (GOF) activities impact multiple hallmarks of cancer cell biology, affecting the chromatin structure, transcriptional regulation, and microRNA biogenesis, shaping the proteome, and rewiring tumor cell metabolic pathways. It also encompasses cytoplasmic functions and cell-extrinsic effects, namely affecting tumor microenvironment and the inflammatory response. Oncogenic GOF of mutp53, driving tumor development and dissemination, relies on the direct interaction with transcription factors (TFs, grey boxes) or co-factors and other protein effectors (yellow boxes), altering their activity, or on the transcriptional modulation of target genes (green boxes). Data retrieved from and based on [8,62,72].

*TP53* mutations in the TAD are associated with the loss of transactivation of specific genes, such as *CDKN1A*, hence blocking the capacity of inducing cell cycle arrest without compromising apoptosis. *TP53* mutations in the OD often halt tetramerization, translating into loss of function (LOF). In addition, *TP53* mutations in the DBD exhibit diverse degrees of functionality and, consequently, different pathological relevance (reviewed in [60]). Besides being primarily associated with LOF [60] (Figure 4A), *TP53* mutations in the DBD commonly occur in a single allele. As such, first-stage tumors are heterozygous, expressing both wt and mutp53. Although wtp53 is still expressed, a dominant negative effect (DNE) [18] of mutp53 over wtp53 is observable and can be explained by the formation of heterotetramers (wtp53 dimer plus mutp53 dimer) devoid of transcriptional activity [60] (Figure 4B). During tumor progression, the loss of heterozygosity is commonly observed and is associated with gain of function (GOF) of sporadic or inherited *TP53* mutations [50] (Figure 4C). GOF can be manifested through mutp53 interaction with diverse transcriptional factors or co-factors. For instance, mutp53 heterooligomerizes with p63 or p73, blocking their tumor suppressor activity by inhibiting their transcriptional activity, or stimulating the transcription of non-canonical genes [61,62]. Mutp53 GOF may also occur through association of mutp53 with C-ets-1/2 (Ets1/2), yes-associated protein (YAP1), peptidyl-prolyl cis-trans isomerase NIMA-interacting 1 (Pin1), or promyelocytic leukemia protein (PML), with transcriptional enhancement of their set of target genes [61,62]. Both scenarios result in altered gene expression patterns that contribute to survival, tumor progression, and more aggressive phenotypes [62]. GOF has been strongly associated with some specific mutp53 forms, such as R175H, R248W, R273H, and R280K (reviewed in [60,62,63]).

It is worth noting that mutp53 is expressed in cancer cells at higher levels than wtp53 in normal cells, indicating that mutp53 is somehow more stable than wtp53 and accumulates in tumor cells [64]. Initially, it was thought that the MDM2 regulatory axis would not work on mutp53 once canonical genes transcription was halted [62]. Nevertheless, MDM2 may be expressed by other pathways [65], and evidence has suggested that mutp53 levels can be controlled by MDM2 in normal tissues, but not in tumor tissues [66]. This raised the possibility of additional events in tumorigenesis responsible for mutp53 accumulation. Indeed, in cancer cells, the occurrence of mutp53 is also associated with increased levels of HSP70 or HSP90, which often bind to and stabilize mutp53 and/or participate in aggregosomes [67]. This impairs the activity of MDM2 or other E3 ligase regulatory proteins, like the carboxy terminus of HSP70-interacting protein [68], culminating in mutp53 accumulation and potentiation of its GOF [53]. Indeed, distinct mutp53 forms have been described as aggregation-prone and are localized in the cytoplasm and perinuclear region rather than in the nucleus as functional wtp53 [69]. These aggregosomes may have a prion-like amyloid behavior [70] and have also been associated with DNE and GOF events by sequestration of wtp53, TAp63, and TAp73. Co-aggregation is possible, since these isoforms share highly conserved aggregating sequences in the same structural motif [69,71].

### Deleterious Effects of Mutant p53 on DNA Binding and Protein Stability

Through the crystallographic model of wtp53 DBD bound to DNA, Cho et al. unveiled the deleterious effects of common cancer mutations [42,45]. Subsequent studies have been pursued to further understand the impact of mutations on p53 structure, stability, and function [45]. In this context, based on the known p53 intrinsic instability, a superstable quadruple mutp53 DBD was developed (M133L, V120A, N239Y, N268D) and used for structural studies (Table 1), enabling its handling without compromising its function [25,73]. These mutations maintain the basic structural scaffold and turn p53 DBD structural framework more rigid, increasing its thermodynamic stability by 2.6 kcal/mol (compared to wtp53 DBD) [25]. The most frequent *TP53* mutations occur in highly conserved sequence regions and coincide with key amino acid residues for DNA recognition and structural stability [74,75] (Figure 2B and Figure 3B). Indeed, single amino acid mutations in the p53 DBD can result in the removal of DNA-contact residues (contact mutp53; e.g., R248Q, R248W, R273H, R273C, R280K) or conformational changes in different parts of the DBD, including structural rearrangements on the DNA-binding surface, creation of internal cavities, or formation of surface crevices in regions remote from the DNA-binding site (structural mutp53; e.g., G245S, G245D, R249S, R175H, Y220C, R282W) [45].

Mutp53 studies by X-ray crystallography and NMR have enlightened local structural changes and their impact on DNA recognition, which are summarized in Table 1. In particular, NMR and in silico simulation data have provided insights into structural changes of L3, with L2 rearrangements, caused by the change of arginine to glutamine in the contact mutp53 R248Q [76,77]. Although structural data is lacking, it is speculated that the substitution of the arginine for a tryptophan in contact mutp53 R248W will abolish the anchoring of p53 to DNA minor grooves due to the hydrophobicity and steric clash of tryptophan, preventing the establishment of hydrogen bonds with DNA [45]. The same is thought to occur in other contact mutp53 forms, such as S241F and C277F [45]. In contact mutp53 R273H and R273C, the histidine and cysteine residues, respectively, alter the hydrogen-bonding network in the DNA-binding surface, compromising the direct contacts to DNA backbone phosphates due to the shorter lateral chains, compared to arginine [24,78,79]. The contact mutp53 R280K exhibits loss of DNA-binding ability with impairment of canonical target genes transcription [80]. Although the switch from arginine to lysine is not markedly different, the lysine is shorter than arginine, and the absence of a guanidinium group affects the p53 DNA-binding ability. It is observed that lysine 280 halts the establishment of essential hydrogen-bonds to the DNA major groove and the contribution of the extended hydrogen-bonding network with other residues [81]. Conformational changes observed in the DNA-binding region of structural mutp53 G245S are small, with the overall conformation conserved [79]. Nevertheless, the serine side chain displaces a structural water molecule, leading to small structural shifts in the proximal residues. This is enough to disturb the protein-protein interface of the dimer, reducing DNA-binding affinity [79]. Conversely, the structural mutp53 R249S leads to a large conformational change of L3, affecting p53 DBD anchoring to the DNA minor groove via R248 contacts [78]. The guanidinium group of the R249 is responsible for stabilizing the L3 hairpin conformation by establishing one salt bridge interaction with D171 and hydrogen bonds with G245 and M246. When the arginine residue changes to serine (R249S), these interactions are abolished. Consequently, L3 acquires more flexibility along with M246 displacement elicited by M243 from its buried location within the hydrophobic zinc-cluster region, leading to the formation of a short *α*-helix. This significant conformational change ultimately displaces R248, halting mutp53 DNA interaction [78]. Additionally, NMR data on mutp53 R249S evidenced an increased flexibility of the *β*-sandwich close to the DNA-binding surface [82].

The residue R282 (H2 *C*-terminal) is responsible for maintaining the structural integrity of the LSH motif by packing H2 to the S2-S2′ *β*-hairpin, anchoring it to the protein core. Through the crystallographic model of structural mutp53 R282W, it was observed that the inclusion of a hydrophobic and bulky tryptophan residue compromised those anchoring interactions. Consequently, the L1 flexibility increases with displacement of the DNA-contact residue K120, affecting DNA recognition [83]. Structural mutp53 Y220C, Y220H, Y220N, and Y220S, although positioned away from the DNA-binding surface, impart deleterious effects on the overall protein stability [19,84]. The mutations occur in the periphery of the *β*-sandwich hydrophobic core, at the beginning of the loop that connects S7 and S8, leading to a mutation-induced crevice on the p53 surface in Y220C, Y220N, and Y220S [79,84]. As the imidazole group of mutant histidine 220 roughly superimposes with the phenol of wt tyrosine, the interactions are partially conserved without observation of crevice formation when comparing with the other Y220 mutants [84]. Other mutations in the *β*-sandwich may occur within its hydrophobic core, such as V143A and F270L. Valine and phenylalanine side chains form an integral part of the hydrophobic core network, and these mutations create internal cavities. Of note, these cavities occur without collapse of the surrounding structure guaranteed by other hydrophobic residues, but they cause a strong destabilization of the core domain [79].

Although no structural data of zinc region mutp53 R175H are available, it is possible to speculate why it abrogates p53 activity. Residue R175 (L2) is embedded between L2 and L3, next to the zinc cluster, and its guanidinium group is responsible for stabilizing this region by establishing hydrogen bonds with P191 and M237 and one salt bridge with D184. If the arginine is replaced by a shorter and bulkier histidine (R175H), structural distortions and interference with zinc binding are expected [45]. Such effects may justify the observed complete loss of DNA-binding ability by R175H [20,85]. In fact, for substitutions bearing smaller side chains, such in the case of R175A (not occurring in cancer), R175C, or R175L, residual binding [20] or wt-like activity [86] can be observed. Conversely, the introduction of large, bulky side chains (e.g., R175W and R175Y) abrogated the wt-like functions in cancer cells [86].

**Table 1:** Impact of mutations on p53 DBD structural features and DNA recognition. Description of main physicochemical amino acid changes that influence the surrounding chemical environment, affecting interactions and structural rearrangements; consequences for mutp53 thermal stability, folding, and DNA-binding ability.

Variable (DBD)	Amino Acid Location and Biochemical Mutation Type	Mutp53 Structural Modifications	Thermo-Dynamic Stability ^&^	Estimated Folded Protein at 37 °C ^#^	DNA-Binding Affinity at 20 °C ^#^	Structural Elucidation of p53 DBD (PDB ID Code Eample)	Ref.
***Wtp53***							
with DNA	*NA*	*NA*	*NA*	*NA*	+++	1TUP *, 1TSR *, 2AC0, 2ADY, 2AHI, 2ATA	[20,36,42]
w/o DNA	*NA*	*NA*	+	+++	*NA*	2OCJ, 1UOL *	[20,87]
***Oncogenic mutp53***							
***DNA contact region***							
S241F	L3; uncharged polar to hydrophobic aromatic/small to large residue	Increase of hydrophobicity and steric clash halts DNA contacts (theoretical interpretation)	*ND*	*ND*	*ND*	*ND*	[45]
R248Q	L3; cationic to uncharged polar residue	Alteration of L3 conformation (NMR data)	*+/−*	+++	−	*ND*	[20,76]
R248W	L3; cationic to hydrophobic aromatic bulky residue	Increase of hydrophobicity and steric clash halts DNA contacts (theoretical interpretation)	*ND*	*ND*	*ND*	*ND*	[45]
R273C	S10; cationic to uncharged polar/large to small residue	Alteration of hydrogen bonds network in DNA-binding surface affecting buttressing residues and DNA contacts	*ND*	*ND*	*ND*	2J20 *, 4IBQ	[24,79]
R273H	S10; aliphatic to aromatic/large to bulky-small residue	Alteration of hydrogen bonds network in DNA-binding surface affecting buttressing residues and DNA contacts	+	+++	−	2BIM *, 4IBS, 4IJT	[20,24,48,78]
C277F	S10-H2 turn; uncharged polar to hydrophobic aromatic/small to large residue	Increase of hydrophobicity and steric clash halts DNA contacts (theoretical interpretation)	*ND*	*ND*	*ND*	*ND*	[45]
R280K	H2; large to small residue	Alteration of hydrogen bonds network in DNA-binding surface affecting buttressing residues and DNA contacts	*ND*	*ND*	−	6FF9	[45,80,81]
***Structural—DNA region***							
F134L	S2′; aromatic to aliphatic residue	*ND*	−	−	−	*ND*	[20]
H168R	L2; aromatic to aliphatic/bulky-small to large residue	Alteration of L2 conformation	*ND*	*ND*	*ND*	2BIN *	[78]
G245S	L3; small to large uncharged polar residue	Small distortion of L3/dimerization interface	+/−	+++	+	2J1Y *	[20,48,79]
G245D	L3; small to large anionic residue	Small distortion of L3 and LSH (*in silico* data)	*ND*	*ND*	*ND*	*ND*	[88]
R249S	L3; cationic to uncharged polar/large to small residue	Alteration of L3 conformation affecting R248-mediated DNA anchoring and of dimer interface; increased flexibility of the *β*-sandwich	+/−	+++	−	2BIO *, 3D05, 3DO6, 3DO7	[20,48,78,82]
R282Q	H2; cationic to uncharged polar residue	Flexibility is decreased in L1 and increased in L3	*ND*	*ND*	*ND*	2PCX	[89]
R282W	H2; aliphatic to aromatic/cationic to hydorphobic bulky residue	Impaired LSH anchoring to *β*-sandwich, increase of L1 flexibility	−	−	++	2J21 *	[20,48,79]
***Structural—Zinc region***							
R175A	L2; cationic to hydrophobic/large to small residue	Interference with zinc-binding (smaller effect than R175H) (theoretical interpretation)	+/−	+++	+	*ND*	[20,45]
R175H	L2; aliphatic to aromatic/large to bulky-small residue	Alteration of L2 and L3 conformation, loss of zinc-binding (theoretical interpretation)	−	−	−	*ND*	[20,45,48]
M237I	L3; large to small residue/decrease in atom electro-negativity	*ND*	−	+/−	−	*ND*	[20]
C242S	L3; decrease in atom electro-negativity	Zinc ligand substitution with loss of zinc coordination (theoretical interpretation)	−	+/−	−	*ND*	[20,45]
***Structural—*β*-sandwich***							
V143A	S3; large to small residue	Internal hydrophobic cavity	−	−	+	2J1W *	[20,48,79]
L145Q	S3; hydrophobic to uncharged polar/small to large residue	β-sheet and loop-sheet-helix motif destabilization (in silico data)	−	+/−	+/−	*ND*	[20,90]
P151S	S3/S4 turn; hydrophobic to uncharged polar residue	*ND*	−	−	+/−	*ND*	[20]
V157F	S4; aliphatic to aromatic/small to large-bulky residue	Internal hydrophobic cavity	−	−	++	4KVP	[91]
I195T	S5; hydrophobic to uncharged polar/large to small residue	*ND*	−	−	−	*ND*	[20]
Y220C	S7–S8 turn; large to small residue	Hydrophobic crevice in the β-sandwich surface at S7–S8 turn	−	−	+/−	2JIX *, 6SHZ *	[20,79,84]
Y220H	S7–S8 turn; hydrophobic to cationic residue	Mild alteration of intermolecular interactions on β-sandwich surface at S7–S8 turn, no crevice observed	*ND*	*ND*	*ND*	6SI1 *	[84]
Y220N	S7–S8 turn; aromatic to aliphatic/bulky hydrophobic to uncharged polar residue	Hydrophobic crevice in the β-sandwich surface at S7–S8 turn	*ND*	*ND*	*ND*	*ND*	[84]
Y220S	S7–S8 turn; aromatic to aliphatic/bulky hydrophobic to small uncharged polar residue	Hydrophobic crevice in the β-sandwich surface at S7–S8 turn	*ND*	*ND*	*ND*	6SI2 *	[84]
I232T	S8; hydrophobic to uncharged polar/large to small residue	*ND*	−	*+/−*	*+*	*ND*	[20]
I255F	S9; aliphatic to aromatic residue	*ND*	−	−	+/−	*ND*	[20]
F270C	S10; hydrophobic aromatic to uncharged polar/large to small residue	Internal hydrophobic cavity (theoretical interpretation)	−	−	+/−	*ND*	[20]
F270L	S10; aromatic to aliphatic/large to small residue	Internal hydrophobic cavity	*ND*	*ND*	*ND*	2J1Z *	[79]

* The protein has four stabilizing mutations (M133L, V203A, N239Y, N268D). ^&^ Difference in free energy of unfolding between wt- and mutp53 DBD at 10 °C ($\Delta \Delta G_{D-N}^{H_{2}O}$), which is extrapolated to 37 °C; free energy of wtp53 used as reference (0 kcal/mol); (+) stable protein ≤ 0.5 kcal/mol, (*+/−*) weakly destabilized protein 0.6–2.5 kcal/mol, (−) strongly destabilized protein ≥ 2.6 kcal/mol. ^#^ Relative folded protein and DNA-binding affinity: (+++) 100–85%, (++) 84–70%, (+) 69–55%, (*+/−*) 54–40%, (−) ≤ 39%. *NA*—not applicable; *ND*—not determined; w/o—without.

Regarding mutp53 stability, Bullock et al. have classified mutp53 DBD in accordance with its thermodynamic and kinetic stability and DNA-binding affinity, relating it to the mutation-specific local structural changes [19,20] (Table 1). The mutp53 stability will dictate the relative amount of folded and potentially functional protein under physiological conditions. It was observed that mutp53 has higher propensity to aggregate than wtp53, with at least 50% of mutp53 denaturated at physiological temperature [20]. For example, the contact mutp53 R273H does not significantly destabilize the DBD, but it halts DNA-binding ability (Table 1), since it is a crucial residue for DNA recognition [20]. Although considered a contact mutp53, R248Q decreases the thermodynamic stability by inducing a structural distortion [20,76,77] (Table 1). The structural distortion induced by mutp53 G245S and R249S slightly reduces protein thermal stability and halts DNA contacts, although G245S retains partial DNA-binding ability at sub-physiological conditions (20 °C) (Table 1). In addition, mutp53 R282W exhibits increased protein destabilization, but it retains partial DNA-binding ability at 20 °C (Table 1), given that most contact residues are undamaged for interaction [20] (Table 1). Similarly, in structural mutp53 Y220C or other *β*-sandwich mutants, despite the conservation of some DNA-binding affinity at 20 °C, the mutations dramatically decrease p53 stability (Table 1) with extensive protein unfolding at physiological temperature [20]. Among the recently described Y220 mutants, Y220H was the least destabilizing; nevertheless, all showed lower melting temperatures than wtp53, similar to Y220C, which suggests that these cancer mutp53 forms are also largely unfolded under physiological conditions [84]. This makes *β*-sandwich structural mutp53 temperature-dependent with respect to its DNA-binding ability [20]. Nevertheless, the same is not verified for the zinc region structural mutp53 R175H, C242S, or M237I, which are extensively denaturated independently of the temperature, thus reinforcing the importance of the zinc ion tethering for the DBD correct folding [20] (Table 1).

It was demonstrated that mutp53 DBD plus OD can lead to misfolding of the tetramer, but not of the monomer. Misfolding appears to involve the intramolecular association of DBD-DBD within the tetramer, promoted by destabilizing mutation in the DBD [21]. In addition, in the case of DNE, although there is a wtp53 dimer, the p53 DBD mutation in mutp53 dimer affects the overall tetramer stability, leading to the loss of binding cooperativity to DNA and subsequent impairment of transcriptional activity [34,35,45,92]. Moreover, mutations in the DBD not only affect DNA recognition, but may also interfere with binding to wtp53 target proteins. For instance, mutp53 forms bearing mutations that do not interfere with L2 and L3 conformation are still capable of interacting with TP53-binding protein 2 (53BP2); however mutp53 G245S impairs this interaction [45]. Importantly, mutp53 cellular half-life is not correlated with thermodynamic stability, as mutp53 tends to accumulate as previously mentioned [53,93].

Despite all the knowledge that has been generated around p53 structure and related topics, the elucidation of how several mutations impact on p53 conformation, stability, and function is still missing.

## 5. Targeting Mutant p53

The restoration of the activity to target proteins poses intrinsic difficulties as a therapeutic strategy when compared to target inhibition [94]. Indeed, regarding mutp53, it could be arguable whether the reestablishment of its tumor suppressor function would be sufficient to counteract a context of multiple oncogenic alterations, including expression of *c*-Myc, GTPase HRas (RAS), or phosphoinositide 3-kinase (PI3K) cancer drivers. However, multiple in vitro and in vivo studies have proven the concept that the reactivation of wt-like function to mutp53 can elicit cell death and halt tumor progression [50,94]. Although mutp53 has long been regarded as an undruggable target [95], some studies have shown that the insertion of artificial second-site mutations can alleviate the effect of inactivating *TP53* mutations [78,96]. The purpose of these rescue mutations is to enable the establishment of new interactions with the DNA, favoring protein stability and correct folding or compensating for missing contacts caused by the original mutated residue [24,26,78,91,96,97,98]. Indeed, this provided the conceptual basis for the feasibility of mutp53 reactivation, by enhancing specific-sequence DNA-binding and transcription of wtp53 target genes. Another positive aspect of mutp53 as a therapeutic target is its high expression levels in cancer cells [99,100].

As formerly evidenced, p53 mutants are not all equal, differing on their structure, stability, and function. Contact mutants, for their structural similarity to wtp53, do not have well-defined hydrophobic pockets to be docked by small molecules [42,95]. Therefore, reactivation of contact mutp53 seems to be a daunting task, since the therapeutic strategy must rely on the introduction of extra interaction points to compensate for the missing DNA contacts [95]. Conversely, structural mutants are found to be kinetically and thermodynamically destabilized in a temperature-dependent (e.g., *β*-sandwich mutations) or temperature-independent (e.g., zinc region mutations) manner. For both structural mutant forms, small molecules acting as chaperones, increasing the level of correctly folded protein at physiological temperature, may be a feasible therapeutic strategy due to the conservation of the DNA-binding residues [20,95].

In the past two decades, consistent efforts from academic and industry research groups have led to the identification of several small molecules (and peptides) that stabilize p53 native conformation and restore sequence-specific DNA binding, rescuing wt-like transcriptional functions and ultimately resulting in cell death and tumor suppression (reviewed in [94,101]) (Table 2). Among these mutp53-targeting agents, two small molecule reactivators have entered clinical trials: PRIMA-1^MET^ (APR-246; Phase III trials; NCT03745716 [102]) and COTI-2 (Phase I trial; NCT02433626 [103]).

Although, some mutp53-targeting agents have shown to bind and stabilize mutp53 (Table 2), the exact mechanism of mutp53 reactivation is still far from being fully understood [94]. The understanding of the precise molecular mechanism underlying the p53 activation of a mutp53-targeting agent, as well as of its off-target effects, is important data for the design of new, more efficient, and safe therapies.

Some of the first molecules reported to activate p53 are Michael acceptors that form adducts by covalently binding to p53 cysteines, causing its stabilization and reactivation [113,119,120,121]. On the other hand, the hydrophobic crevice suitable for binding exhibited by mutp53 Y220C prompted the design of several molecules able to reactivate its wt-like function [130,131,133]. Notably, the affinity of Y220C-cavity binders has progressively improved since PhiKan083 was developed, and further optimizations may be predicted [132]. Furthermore, in silico studies have identified new druggable p53 regions, namely a transiently open putative binding pocket in L1/S3 of the p53 DBD, in its folded conformation. This pocket is suitable to accommodate small molecules, thermodynamically stabilizing p53 in its native conformation and reactivating its transcriptional function [140]. The targeted therapy using zinc metallochaperones represents a unique approach, which is not based on a direct protein-ligand interaction, but instead on increasing the intracellular levels of zinc that are appropriate for remetallating and refolding of conformational mutp53 DBD [125]. A different approach targeting the DNA-binding surface of mutp53 DBD describes a chaperone mechanism (through non-covalent binding) by peptides or small molecules for the rescuing of the unfolded mutp53, shifting the equilibrium towards the native state and then being displaced in the presence of DNA [142,144]. In this regard, in silico results denoted a distinct molecular mechanism of mutp53 reactivation by SLMP53-1 and MANIO, not dependent on covalent bonds or mutation-created hydrophobic pockets, as described for other mutp53 reactivators. SLMP53-1 and MANIO reactivates mutp53 R280K and R248W, respectively, through binding to a hydrophobic pocket of the protein homodimer, establishing an interface with the DNA minor groove, and compensating for the loss of direct contacts between the K280/W248 residues and the DNA. Interestingly, the superposition of wt- and mutp53 models showed that the compounds have a similar binding pattern to wtp53, which is consistent with the reported activation of wtp53 by SLMP53-1 and MANIO [135,138]. It is also interesting to note that these compounds can reactivate other mutp53 forms, both contact and structural, which further supports a molecular mechanism unrelated to the formation of pockets derived from specific mutation sites.

An alternative mutp53-targeting approach includes disruptors of protein-protein interaction, which inhibit the interaction of mutp53 with other proteins, thereby suppressing mutp53 GOF activities (Table 2). The small molecule LEM2, besides inducing thermal stability to p73, also halted the interaction of mutp53 and p73, leading to tumor suppression by the transcription of p53-shared target genes [153]. Moreover, ReACp53, a mutp53 targeting peptide, has shown the capability to inhibit mutp53 containing aggregosomes by binding to S9 (an aggregation prone region) and favoring wt-like folding to structural mutp53 forms [156]. In silico studies disclosed that mutp53 forms exhibit an “open” state of the S6-S7 turn, exposing S9, and therefore representing a putative binding pocket to be explored in drug design [170].

Mutp53 protein degradation by small molecules, rather than its reactivation, with observed cellular growth inhibitory effect, constitutes another mutp53 targeting strategy [67] (Table 2). This strategy could also be explored through a novel pharmacological approach, such as PROteolysis TArgeting Chimeras (PROTACs) [171]. For this, the combined knowledge of mutp53 structure and putative binding pockets or surfaces, known mutp53-targeting agents, and small molecules that bind to E3 ligases may boost the discovery of effective therapeutic options.

Although none of the reported mutp53-targeting compounds have reached the clinic, the structural and mechanistic diversity among them is encouraging. It is reasonable to expect that some of them will prove effective in clinical applications in the near future [94]. Furthermore, the pharmacological reactivation of mutp53 also poses a great opportunity in combination therapy with anticancer drugs known to trigger p53-dependent cancer cell death or to inhibit mutp53 downstream pathways [94].

However, it has to be considered that although the potential of reactivating wt-like functions of mutp53 with canonical gene transcription has been proven, this does not always translate into cell death or halt tumor growth, as this also depends on the cancer cell context [58,94].

## 6. Conclusions

Structural studies have strongly contributed to knowledge about p53 stability, its behavior in the presence of inhibitors or co-factors, and how the protein recognizes and binds to target DNA sequences, thereby inducing coordinated changes in gene transcription within its vast and complex regulatory network. Furthermore, they have provided insights into wt and mutp53 pharmacology at molecular levels, fostering the design and development of p53-based targeted therapies to halt cancer. However, despite all the strategies detailed above to target mutp53, drugging mutp53 still prompts researchers and clinicians to investigate new possibilities in the universe of mutp53 pharmacology, structure, and biology.

Concerning the structural elucidation of mutp53, it would be valuable to further understand the structural and functional differences of other mutp53 forms. In particular, the classical categorization of mutp53 into contact and structural forms, and the possibility of reactivating each class by establishing extra contacts to DNA or by increasing thermal stability [95,120] seem to be inadequate based on the reported data for mutp53 reactivators. In fact, many mutp53 reactivators are capable of reactivating both contact and structural mutp53 with restoration of DNA-binding and transcriptional activity. Some of them have even induced the thermal stability of both mutp53 classes to increase the fraction of wt-like conformation [49,108,111,121,139,143]. Other important issues to be further explored are the mechanisms underlying the mutp53 aggregation-prone ability [83,156,172,173], the study of mutp53 interactomes, the mapping of potential PTMs on mutp53, and the impact of small molecules in these events. All such studies would greatly contribute towards a better understanding of mutp53 pathobiology and pharmacology, galvanizing the discovery and development of new mutp53 targeting agents for personalized cancer therapy.

## Figures and Tables

**Table 2:** Summary of reported mutp53-targeting agents. Agents are organized by their chemical class, discovery strategy, targeted mutp53 forms, mechanism of action, and main biophysical and cellular outcomes.

Chemical Name (Class)	Discovery Strategy	Targeted * Mutp53	Mechanism of Action	Observations	Ref.
***Reactivators—Cysteine-targeting alkylation***					
**CP-31398** (Styrylquinazoline) 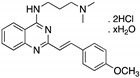	Chemical library; protein-based screening assay	V173A; S241F; R249S; R273H	Michael addition ^#^ Binding: *ND*	Transcription of p53 target genes; p53-dependent and -independent in vitro antitumor activity	[104,105,106,107]
**MIRA-1** (Maleimide) 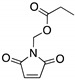	Chemical library; cellular screening assay	R175H; P176Y/R248W; R248Q; R248W; R273H; R273H/P309S; R280K; R282W	Michael addition ^#^ Binding: *ND*	Rescue of wt conformation to mutp53 R248W, R175H; restoration of DNA binding to mutp53 R175H, R248Q, P176Y/R248W, R280K, R282W; restoration of transcriptional activity to mutp53 R175H, R273H, R273H/P309S; p53-dependent and -independent in vitro and in vivo antitumor activity	[108]
**STIMA-1** (Styrylquinazoline) 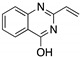	Chemical library; cellular screening assay	R175H; R273H	Michael addition ^#^ Binding: *ND*	Restoration of DNA binding to mutp53 R175H; restoration of transcriptional activity to mutp53 R175H, R273H; p53-dependent and -independent in vitro antitumor activity	[107]
**KSS-9** (Piperlongumine derivative) 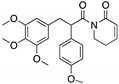	Rational design	R175H	Michael addition ^#^ Binding: *ND*	Rescue of wt conformation; restoration of DNA binding to mutp53; transcription of p53 target genes; p53-dependent and -independent in vitro antitumor activity	[109]
**PRIMA-1** **PRIMA-1^MET^** (Quinuclidinone) 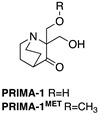	Chemical library; cellular screening assay	R175H; R273H; D259Y/K286E; K286E; S241F; R273C; P223L/V274F	Metabolized to methylene quinuclidinone (active metabolite), Michael addition Binding: mutp53 R175H, R273H	Enhanced thermal stability of wtp53, mutp53 R175H, R273H; rescue of wt conformation to mutp53 R175H; restoration of transcriptional activity to mutp53 R175H, R273H, D259Y/K286E, K286E, S241F, R273C, P223L/V274F; p53-dependent and -independent in vitro and in vivo antitumor activity	[110,111,112,113,114,115,116,117,118,119]
**PK11007** (Sulfonylpyrimidine) 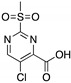	Chemical library; protein thermal stability-based screening assay	Y220C; V143A	Binds p53 by nucleophilic aromatic substitution Binding: mutp53 Y220C	Enhanced thermal stability of mutp53; transcription of p53 target genes; p53-dependent and -independent in vitro antitumor activity	[120]
**HO-3867** (Diarylidenyl piperidone curcumin analogue) 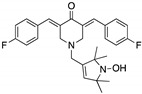	Chemical library; cellular screening assay	K132Q; R156P; Y163H; R175H; H193R; L194F; Y205F; P223L/V274F; C238Y; N239D; S241F; G245S; G245V; M246I; R248Q; R248W; R249S; R273H; C277F; R280K; E285K	Binds p53 by Michael addition Binding: mutp53 Y220C	Rescue of wt conformation to mutp53 P223L/V274F, R273H, R280K; restoration of transcriptional activity to mutp53 K132Q, R156P, Y163H, R175H, H193R, L194F, Y205F, C238Y, N239D, S241F, G245S, G245V, M246I, R248W, R248Q, R249S, R273H, C277F, R280K, E285K; p53-dependent in vitro and in vivo antitumor activity	[121,122]
***Thermal stabilizers***					
**3-Benzoylacrylic acid** (Benzoylacrylate) 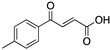	Chemical library; protein thermal stability-based screening assay	Y220C; R175H; G245D; R249S; R282W	Michael addition Binding: mutp53 Y220C	Enhanced thermal stability of mutp53 Y220C, R175H, G245D, R249S, R282W; absence of in vitro antitumor activity evaluation	[123]
***Reactivators—Zinc chelators***					
**ZMC1/NSC319726** (Thiosemicarbazone) 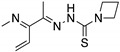	Database analysis; cellular screening assay	R175H; C176F; C238S; C242S; C242F; G245S	Zn^2+^ chelator Binding: mutp53 R175H	Increased cellular zinc concentration; rescue of wt conformation to mutp53 R175H, C176F, C238S, C242S; restoration of transcriptional activity to mutp53 R175H, C176F, C238S, C242S, G245S; p53-dependent and -independent in vitro and in vivo antitumor activity	[124,125]
**COTI-2** (Thiosemicarbazone) 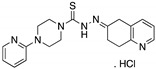	Rational drug design; virtual drug screening	R175H	Zn^2+^ chelator Binding: *ND*	p53-dependent and -independent in vitro and in vivo antitumor activity	[126,127,128]
**Benzothiazolyl, Benzoxazolyl Hydrazones** (C85) 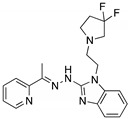	Chemical library; biophysical and cellular screening assay	R175H	Zn^2+^ chelator Binding: *ND*	Increased cellular zinc concentration; rescue of wt conformation to mutp53 R175H, zinc-deficient p53-dependent in vitro and in vivo antitumor activity	[129]
***Reactivators—Non-covalent binding***					
**PK083 and analogs** (Carbazole) 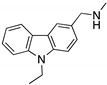	Rational drug design; virtual drug screening	Y220C, Y220N, Y220S	Binds to a cleft in *C*-terminal of mutp53 Y220C, Y220N, and Y220S DBD	Enhanced thermal stability (Y220C, Y220N, Y200S); rescue of wt conformation to mutp53 (PK083-Y220C); inhibition of aggregation (PK9318 analog-Y220C); transcription of p53 target genes (PK9318 analog-Y220C); p53-dependent in vitro antitumor activity	[84,130,131,132]
**PK7088** (Pyrazole) 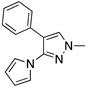	Rational drug design; NMR protein-based screening assay	Y220C	Binds to a cleft in *C*-terminal of mutp53 Y220C DBD	Enhanced thermal stability; rescue of wt conformation to mutp53; transcription of p53 target genes; weak in vitro antitumor activity	[131,133]
**PK5196** (Halogen-phenol derivative) 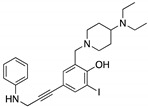	Rational drug design; NMR protein-based screening assay	Y220C	Binds to a cleft in *C*-terminal of mutp53 Y220C DBD	Enhanced thermal stability; in vitro antitumor activity	[131,134]
**MB725** (Aminobenzothiazole) 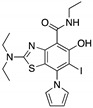	Rational drug design; NMR protein-based screening assay	Y220C	Binds to a cleft in *C*-terminal of mutp53 Y220C DBD	Enhanced thermal stability; transcription of p53 target genes; p53-dependent in vitro antitumor activity	[131]
**SLMP53-1** (Tryptophan-derived isoindolinone) 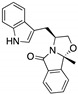	Chemical library; yeast-targeted screening assay	R175H, G245D, R248Q, R248W, R273H, R280K, R282W	Binds to mutp53 R280K DBD; in silico proposes that SLMP53-1 bridges DNA-binding surface of mutp53 R280K to DNA minor groove	Enhanced thermal stability of wt- and mutp53 R280K; restoration of DNA binding to mutp53 R280K; restoration of transcriptional activity to mutp53 R280K; p53-dependent in vitro and in vivo antitumor activity	[135,136]
**HBAP** (2-[(4-hydroxybenzyl) amino]phenol) 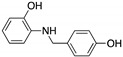	Chemical library; cellular screening assay	R280K; R273H	Binds to mutp53 R280K and R273H DBD	Transcription of p53 target genes; in vitro and in vivo antitumor activity	[137]
**MANIO** (Thiazole derivative) 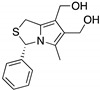	Chemical library; cellular screening assay	Y126C; R175H; G245D; G245S; R248Q; R248W; R280K; R282W; R273C; R273H	Binds to wt- and mutp53 R248W DBD; in silico proposes that MANIO fits between the DNA molecule and the protein pocket at the dimer interface	Enhanced thermal stability of wt- and mutp53 R248W, Y126C, and R273H; increase of protein DNA-binding ability; transcription of p53 target genes; p53-dependent in vitro and in vivo antitumor activity	[138]
**SCH529074** (Piperazinylquinazoline) 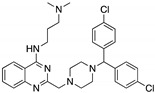	Chemical library; DNA-binding assay	R175H; S241F; R248W; R249S; R273H	Binds to wtp53 nearby DNA binding surface	Protected wtp53 conformation from thermal denaturation; rescue of wt conformation to mutp53 S241F, R248W, R273H; restoration of DNA binding to mutp53 R175H, R249S, R273H; transcription of p53 target genes by mutp53s R175H, S241F R248W, R249S, R273H; blocked MDM2-mediated ubiquitination of p53; p53-dependent in vitro and in vivo antitumor activity	[139]
**Stictic acid** 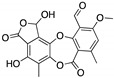	Chemical library; virtual screening	R175H; G245S	In silico binding to wtp53 and mutp53 R175H, R273H, G245S to a transiently open pocket (L1/S3)	Enhanced thermal stability of mutp53 R175H, G245S; transcription of p53 target genes by mutp53 R175H, G245S; p53-dependent in vitro antitumor activity	[140]
**Curcumin, Flavokawain B, Alpinetin** 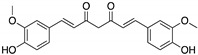 **Curcumin** 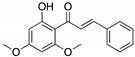 **Flavokawain B** 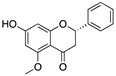 **Alpinetin**	Crude extracts; cellular screening assay	R273H	In silico binding to mutp53 R273H bridging DNA-binding surface to DNA sequence	p53-dependent and -independent in vitro antitumor activity	[141]
**pCAPs** (Peptides) pCAP221 sequence: RRKHNKHRPEPDSDER pCAP242 sequence: RRLIVRILKLPNPPER pCAP250 sequence: RRHSTPHPD	Phage peptide display-protein screening assay	V135A; S241F; R249S; R280K	Binding to unknown local of mutp53 R175H and R249S	Rescue of wt conformation to mutp53 R175H, R249S; restoration of DNA binding to mutp53 R175H, R249S; transcription of p53 target genes by mutp53 V135A, S241F, R280K; p53-dependent in vitro and in vivo antitumor activity	[142]
**CDB3** (Peptide) Sequence: REDEDEIEW	Rational drug design; NMR protein-based screening assay	R175H; I195T; R249S; R273H	Binding to wt and mutp53 R249S, R273H, nearby DNA binding surface and G245S, R175H unknown local	Enhanced thermal stability of wt and mutp53s R175H, R249S, R273H; rescue of wt conformation to mutp53 R175H, R249S, R273H; enhanced DNA binding to mutp53 I195T; transcription of p53 target genes by mutp53 R175H, R273H; p53-dependent in vitro antitumor activity	[82,143,144]
***Reactivators—Chaperone-mediated effect***					
**Chetomin** (Epidithiodioxopiperazine) 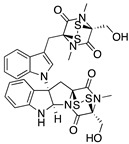	Natural products database; cellular luciferase reporter screening assay	R175H	Binds to HSP40	Rescue of wt conformation to mutp53; transcription of p53 target genes; MDM2 negative regulation; p53-dependent and -independent in vitro and in vivo antitumor activity	[145]
**SLMP53-2** (Tryptophanol-derived oxazoloisoindolinone) 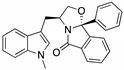	Chemical library; cellular screening assay	Y220C	Enhances the mutp53 Y220C interaction with HSP70	Rescue of wt conformation to mutp53; transcription of p53 target genes; p53-dependent in vitro and in vivo antitumor activity	[146]
***Reactivators—Unknown binding***					
**Ellipticine** (Alkaloid) 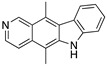	Chemical library; cellular screening assay	R175H; L194F; S241F; R249S; R273C; R273H	*ND*	Restoration of DNA binding to mutp53 R175H, S241F; rescue of wt conformation to mutp53 S241F; restoration of transcriptional activity to mup53 R175H; L194F, S241F, R249S, R273C, R273H; p53-dependent in vitro antitumor activity	[147]
**P53R3** (Piperazinylquinazoline) 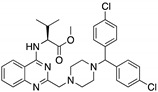	Chemical library; DNA-binding assay	M237I; R175H; R273H R248W	*ND*	Restoration of DNA binding to mutp53 R175H, M237I, R273H; restoration of transcriptional activity to mup53 M237I; p53-dependent in vitro antitumor activity	[148]
**PEITC** (Phenethyl isothiocyanate) 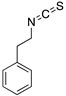	Cellular screening assay	R175H	*ND*	Rescue of wtp53 conformation to mutp53; transcription of p53 target genes; p53-dependent in vitro and in vivo antitumor activity	[149]
**WR-1065** (Aminothiol) 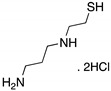	Active metabolite of amifostine; cellular screening assay	V272M	*ND*	Restoration of DNA binding; rescue of wt conformation to mutp53; transcription of p53 target genes; p53-dependent and -independent in vitro antitumor activity	[150]
***Disruptors of protein-protein interaction***					
**RETRA** (Thiazolthiophenyl ethanone) 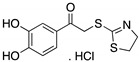	Chemical library; cellular screening assay	R273H	Disrupts mutp53-TAp73 complexes	Increased TAp73 expression; transcription of p53-shared target genes; TAp73-dependent in vitro and in vivo antitumor activity	[151]
**Prodigiosin** (Pyrrolyl pyrromethane) 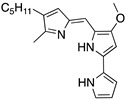	Chemical library; cellular screening assay	R273H; S241F; R248Q	Disrupts mutp53-p73 complexes	Transcription of p53-shared target genes; p73-dependent in vitro antitumor activity	[152]
**LEM2** (Xanthone) 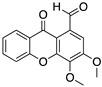	Chemical library; yeast-targeted screening assay	R273H	Disrupts mutp53-TAp73 and MDM2-TAp73 complexes	Enhanced thermal stability of TAp73; transcription of TAp73- and p53-shared target genes; TAp73-dependent in vitro antitumor activity	[153]
**Statins** (Lovastatin) * 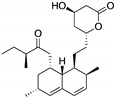 *	Cellular screening assay	R156P; V157F; R175H; Y220C; R248W	Inhibition of the mevalonate pathway, with CHIP-mediated mutp53 degradation	In vitro and in vivo suppression of mutp53-expressing cancer cell growth	[154]
**ATRA** (Retinoic acid; tretinoin) 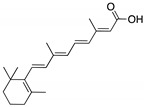	Chemical library; mechanism-based screening assay (protein active site)	R273H; R280K	Disrupts mutp53-Pin1 interaction (via Pin1 inhibition and degradation)	In vitro and in vivo antitumor activity	[155]
**ReACp53** (Peptide) Sequence: RRRRRRRRRRPILTRITLE	Structure-based rational design; cellular screening assay	R175H; R248Q	Binds to mutp53 aggregation prone region (S9)	Inhibits mutp53 aggregates; shifts the folding equilibrium toward the wt conformation; transcription of p53 target genes; p53-dependent in vitro and in vivo antitumor activity	[156]
***Inducers of mutp53 degradation***					
**17-AAG; 17-DMAG** (Demethoxygeldanamycin derivatives) 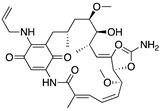 17-AAG 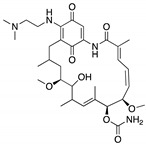 17-DMAG	HSP90 inhibitor; cellular evaluation	L194F; R273H; R273H/P309S; R280K	Inhibits HSP90 with increase of MDM2 and CHIP function	In vivo antitumor activity in synergism with SAHA	[157,158]
**Geldanamycin** (Benzoquinone ansanamycin) 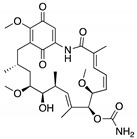	HSP90 inhibitor; cellular evaluation	R175H; L194F; R248Q; R273H; R280K; R172H (mouse)	Inhibits HSP90 with increase of MDM2 and CHIP function	In vitro antitumor activity	[159,160]
**Ganetespib** (Phenylindolyl triazolone) 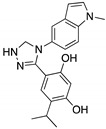	HSP90 inhibitor; cellular evaluation	C124R; R172H; L194F; S241F; R248Q; R273H; C275F	Inhibits HSP90 with mutp53 degradation	In vivo and in vitro antitumor activity	[158]
**SAHA** (Suberoyl-anilide hydroxamic acid) 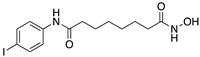	HDAC inhibitor; cellular evaluation	L194F; P223L/V274F; R249S; R273H; R273H/P309S; R280K	Inhibits HDAC6/8 (HSP90 machinery) with mutp53 CHIP-ubiquitin/proteasome-mediated degradation; decreases association with YY-1 transcription factor halting GOF	In vivo and in vitro antitumor activity	[158,161,162,163]
**Sodium butyrate** 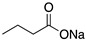	HDAC inhibitor; cellular evaluation	R249S; R280K	Inhibits HDAC8 (HSP90 machinery) with decreased association with YY-1 transcription factor halting GOF	In vitro antitumor activity	[163]
**Arsenic trioxide** 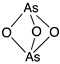	Cellular evaluation	R175H; H179Y/R282W; R248W; R270H; R273H; R273H/P309S	Induces mutp53 nuclear proteasome-mediated degradation	Besides inducing mutp53 degradation, arsenic compounds stabilized wtp53 levels in cancer cells; in vitro antitumor activity	[164]
**Gambogic acid** (Xanthone) 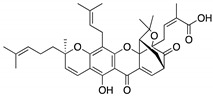	Cellular evaluation	R175H; G266E; R273H; R280K	Depletes mutp53 via HSP90-CHIP ubiquitin/proteasome-mediated degradation	In vitro antitumor activity	[165]
**Spautin-1** (Fluorobenzylquinazolin amine) 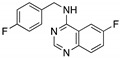	USP inhibitor; cellular evaluation	P98S; P151H; S158inF; A161T; R175C/D/H; L194F; S227K/R; S241F; G245C; R248L/Q/W; E258K; G266E; R273H/L; R280K; R282W	Inhibits deubiquitinating enzymes leading to mutp53 lysosome-mediated degradation	In vitro antitumor activity	[166]
**YK-3-237** (Chalcone) 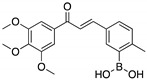	Chemical library; cellular evaluation	V157F; M237I; R249S; R273H; R280K	Activates deacetylase SIRT1 reducing p53 levels	Transcription of p53 target genes; p53-dependent in vitro antitumor activity	[167]
**NSC59984** (Methylpiperazinylnitrofuranyl propenone) 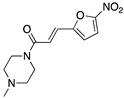	Chemical library; p53-reporter gene cellular screen	R175H/L; S241F; R273H/P309F	Induces MDM2-ubiquitin-proteasome-mediated degradation	Transcription of p53 target genes; p73-dependent in vitro and in vivo antitumor activity	[168]
**Disulfiram** (Tetraethylthiuram disulfide) 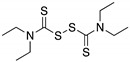	Cellular evaluation	R273H	Thiol-conjugation with mutp53 proteasome-mediated degradation	In vivo and in vitro antitumor activity	[169]

^#^ The compound is a Michael acceptor, suggesting that the mechanism of action occurs by Michael addition to p53 cysteine-thiol groups; nevertheless, the reaction was not determined. *ND*—not determined. CHIP—E3 ubiquitin-protein ligase CHIP; HDAC—histone deacetylase; HSP—heat shock protein; Pin1—peptidyl-prolyl cis-trans isomerase NIMA-interacting 1; SIRT-1—NAD-dependent protein deacetylase sirtuin-1; USP—ubiquitin carboxyl-terminal hydrolase; YY-1—transcriptional repressor protein YY1. * Targeted mutp53 by small molecules through mechanisms of reactivation of wt-like p53 activities, disruption of mutp53 interaction with other proteins, or protein degradation.
